# Mass production-enabled computational spectrometers based on multilayer thin films

**DOI:** 10.1038/s41598-022-08037-y

**Published:** 2022-03-08

**Authors:** Cheolsun Kim, Pavel Ni, Kang Ryeol Lee, Heung-No Lee

**Affiliations:** 1 School of Electrical Engineering and Computer Science, Gwangju Institute of Science and Technology, Gwangju, 61005 South Korea; 2 SNI Co., Ltd., Seongnam, Gyeonggi 13509 South Korea

**Keywords:** Mathematics and computing, Optics and photonics

## Abstract

Multilayer thin film (MTF) filter arrays for computational spectroscopy are fabricated using stencil lithography. The MTF filter array is a 6 × 6 square grid, and 169 identical arrays are fabricated on a single wafer. A computational spectrometer is formed by attaching the MTF filter array on a complementary metal–oxide–semiconductor (CMOS) image sensor. With a single exposure, 36 unique intensities of incident light are collected. The spectrum of the incident light is recovered using collected intensities and numerical optimization techniques. Varied light sources in the wavelength range of 500 to 849 nm are recovered with a spacing of 1 nm. The reconstructed spectra are a good match with the reference spectra, measured by a grating-based spectrometer. We also demonstrate computational pinhole spectral imaging using the MTF filter array. Adapting a spectral scanning method, we collect 36 monochromatic filtered images and reconstructed 350 monochromatic images in the wavelength range of 500 to 849 nm, with a spacing of 1 nm. These computational spectrometers could be useful for various applications that require compact size, high resolution, and wide working range.

## Introduction

Spectrometers are powerful tools for remote sensing and medical applications^1–3^. However, these uses are restricted to research and development due to limitations based on the spectrometers’ bulky size, high cost, and long measuring time. There have been tremendous efforts to overcome spectrometer limitations and go beyond restricted applications^4–21^. One promising candidate to achieve this is optical filter array-based spectrometers: complementary metal–oxide–semiconductor (CMOS) image sensors with a filter array used as a spectrometer. These spectrometers are smaller and have faster measuring times, so they are useful in portable applications, such as on-site detection and small unmanned aerial vehicle (UAV)-based remote sensing. However, the number of filters that can be attached to a CMOS image sensor is limited due to its small sensing area. Thus, these spectrometers offer a low spectral resolution.

Over the past decade, computational approaches^22,23^ have been adapted for filter-based spectrometers. The spectral resolution in conventional filter array-based spectrometers  has been improved using computational approaches. New optical filter types have been proposed that work well in computational approaches and achieve further improvements^6–8,10,11,13,14,16,21^. Unlike conventional optical filters, which selectively transmit incident light in specific wavelengths and reflect the remaining wavelengths, these filters, called random spectral filters, modulate and transmit incident light with unique spectral features in the entire wavelength ranges of interest. Various types of random spectral filters have been proposed, such as etalon filters^10,11,20^, quantum dot filters^8,21^, photonic crystal slabs^7,9,14,16^, and multilayer thin films (MTF) filters^6,13^. The spectral resolvability of computational spectroscopy has been successfully demonstrated using random spectral filters with low correlation among filters.

In contrast to transmission functions of an etalon filter, which consists of repetitive narrow peaks, and a quantum dot filter, which consists of a broadband peak, the fabricated random spectral filter has a transmission function of multiple peaks with various full widths at half maximums (FWHMs) and has a large difference between maximal and minimal transmission in the transmission function. By utilizing the computational approaches, a wide wavelength range can be covered with a small number of MTF filters. In this work, a small number of MTF-based random spectral filters were fabricated in the form of an array. 169 identical filter arrays, consisting of 36 MTF filters, were fabricated on a single wafer. We realized MTF filters by stacking multiple layers of two alternating materials with high and low refractive indices. Using stencil lithography based on shadow masks, we could fabricate MTF filters with different spectral features simultaneously as a filter array form. This idea of random spectral filters can be applied to various wavelength ranges by changing the MTFs’ design properties. According to the usage of MTF filters, they can also be mass-produced in various shapes using stencil lithography techniques.

Here, we demonstrate the competence of spectral reconstructions over the wavelength range of 500 to 849 nm using a fabricated filter array. The fabricated filter array consists of 36 MTF filters in the shape of a square. Varied spectra of incident light such as monochromatic, broadband, and continuous light is used to test the filter array’s reconstruction performances. Additionally, we perform pinhole spectral imaging using the filter array, showing that computational spectral imaging is possible.

## Results

### Spectrometers based on MTF filters

**Figure 1:**
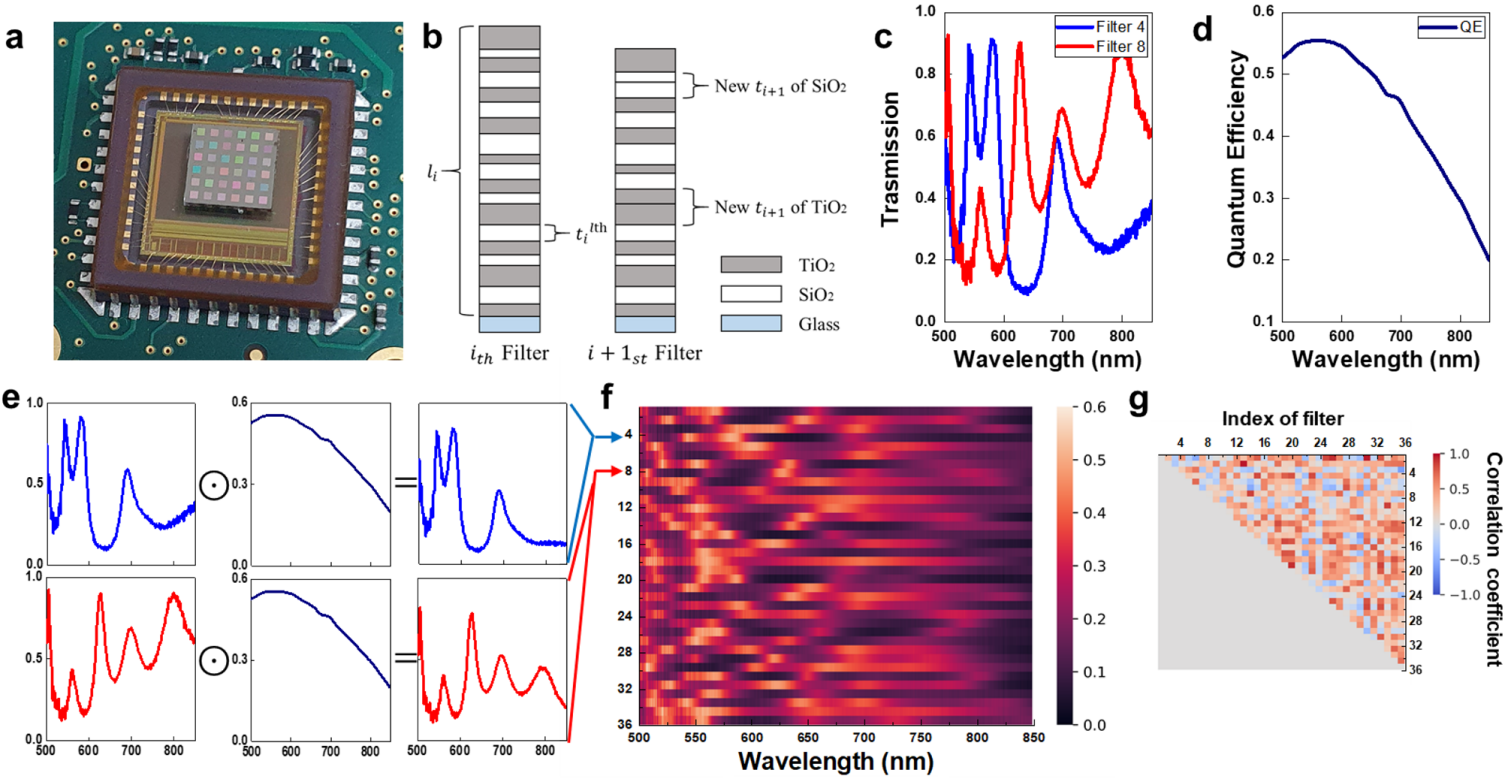
Multilayer thin films (MTF) based computational spectrometer. (**a**) Photograph of the MTF filter array, which is directly attached to the CMOS image sensor. (**b**) Schematic of MTF filters: TiO_2_ and SiO_2_ are deposited on a glass substrate with design properties such as the number of layers of the *i*-th MTF filter $\left( {l_{i} } \right)$ and thickness of the *l*-th layer of the *i*-th MTF filter $\left( {t_{i}^{{l{\text{th}}}} } \right).$ (**c**) Two transmission functions of MTF filters. (**d**) Spectral response of the CMOS image sensor. (**e**) Spectral sensitivity of an MTF filter with the CMOS image sensor, which can be calibrated by element-wise multiplication of the transmission function and the spectral response of the CMOS image sensor. (**f**) Heatmap of the sensing matrix. Each row represents the spectral sensitivity with respect to wavelength. (**g**) Upper triangular matrix of correlation coefficients which are pairwise compared among rows of the sensing matrix.

The computational spectrometer consists of MTF filters and the CMOS image camera. As shown in Fig. 1a, MTF filters are in the form of an array and are directly attached to the CMOS image sensor. Each filter has unique spectral features that can be realized by stacking multiple layers of thin films. A schematic of the MTF filter is shown in Fig. 1b. The transmission function of the *i*-th MTF filter is determined by design properties, such as the number of layers $\left( {l_{i} } \right)$ and the thickness of the *l*-th layer $\left( {t_{i}^{{l{\text{th}}}} } \right).$ Using the transfer-matrix method, the transmission function of the MTF filter can be calculated^24,25^. We choose a set of different design properties to produce a set of filters with a unique transmission function. Let us denote the transmission function of the *i*-th MTF filter in the wavelength range ${{\varvec{\uplambda}}} = \left[ {\lambda_{1} ,\lambda_{2} , \ldots ,\lambda_{N} } \right]$ as ${\mathbf{T}}_{i} = \left[ {T_{i} \left( {\lambda_{1} } \right),T_{i} \left( {\lambda_{2} } \right), \ldots ,T_{i} \left( {\lambda_{N} } \right)} \right].$ Figure 1c shows two measured transmission functions of the MTF filters (see Sect. 4 for measuring transmission functions of MTF filters). The intensity, $y_{i}$, measured by CMOS image sensor for an unknown incident spectrum ${\mathbf{x}} = \left[ {x\left( {\lambda_{1} } \right),x\left( {\lambda_{2} } \right), \cdots ,x\left( {\lambda_{N} } \right)} \right]^{T} ,$ can be expressed as:

1 $$y_{i} = \sum\limits_{k = 1}^{N} {T_{i} \left( {\lambda_{k} } \right)Q\left( {\lambda_{k} } \right)x\left( {\lambda_{k} } \right)} ,$$

where ${\mathbf{Q}} = \left[ {Q\left( {\lambda_{1} } \right),Q\left( {\lambda_{2} } \right), \ldots ,Q\left( {\lambda_{N} } \right)} \right]$ is the spectral response of CMOS image sensor in the wavelength range ${{\varvec{\uplambda}}}.$ The spectral response is represented in Fig. 1d. Let us set $R_{i} \left( {\lambda_{k} } \right) = T_{i} \left( {\lambda_{k} } \right)Q\left( {\lambda_{k} } \right),$ where $R_{i} \left( {\lambda_{k} } \right)$ represents spectral sensitivity of the *i*-th filter of the CMOS image sensor at the wavelength $\lambda_{k} ,$ the Eq. (1) becomes $y_{i} = \sum\nolimits_{k = 1}^{N} {R_{i} \left( {\lambda_{k} } \right)Q\left( {\lambda_{k} } \right)} .$ Considering an *M* number of filters, there is a set of *M* equations for $i = 1,2, \ldots ,M.$ The set of *M* equations can be represented in matrix formation:

2 $$\left[ {\begin{array}{*{20}c} {y_{1} } \\ \vdots \\ {y_{M} } \\ \end{array} } \right] = \left[ {\begin{array}{*{20}c} {R_{1} \left( {\lambda_{1} } \right)} & \cdots & {R_{1} \left( {\lambda_{N} } \right)} \\ \vdots & \ddots & \vdots \\ {R_{M} \left( {\lambda_{1} } \right)} & \cdots & {R_{M} \left( {\lambda_{N} } \right)} \\ \end{array} } \right]\left[ {\begin{array}{*{20}c} {x\left( {\lambda_{1} } \right)} \\ \vdots \\ {x\left( {\lambda_{N} } \right)} \\ \end{array} } \right],$$

where ${\mathbf{y}} \in {\mathbb{R}}^{M \times 1}$ is a column vector with measured intensities from *M* filters and ${\mathbf{R}} \in {\mathbb{R}}^{M \times N}$ is the sensing matrix where each row represents the spectral sensitivity with respect to the wavelength. The spectral sensitivity can be calibrated by element-wise multiplication of the transmission functions of MTF filters and the spectral response of the CMOS image sensor, as depicted in Fig. 1e,f.

Conventional spectrometers read out ${\mathbf{y}}$ as the incident spectrum ${\mathbf{x}}.$ In order to make the measured intensities ${\mathbf{y}}$ as close as possible to the incident spectrum ${\mathbf{x}},$ the sensing matrix ${\mathbf{R}}$ should be an identity matrix with the dimension of $N \times N(M = N).$ This means that *N* number of filters are needed in conventional manners. In practice, it may be difficult to fabricate a narrow FWHM filter and, since the number of filters required increases as the wavelength of interest increases, it is more challenging to make a compact spectrometer operating in a wide wavelength range. Unlike conventional filter-based spectrometers, computational spectrometers modulate and measure a wide wavelength range of the incident spectrum using a small number of MTF filters. We consider the sensing matrix ${\mathbf{R}}$ with dimensions $M \times N(M < N).$ The set of *M* equations becomes an underdetermined problem. Reconstruction algorithms^26–28^ can be applied to restore the incident spectrum in high resolution by solving the underdetermined problem.

Figure 1f shows the heatmap of the sensing matrix of the fabricated MTF filter array-based computational spectrometer. Each row represents the spectral sensitivity with respect to wavelength. The correlation coefficients for each pair of two rows of the sensing matrix are shown as the upper triangular matrix in Fig. 1g. The average value of the correlation coefficients is 0.231, which can be described as a weak or moderate correlation among sensitivities. With the weakly correlated spectral sensitivities, the incident spectrum was measured as unique intensities, which allow the reconstruction algorithms to work effectively.

### Fabrication of MTF filter arrays

**Figure 2:**
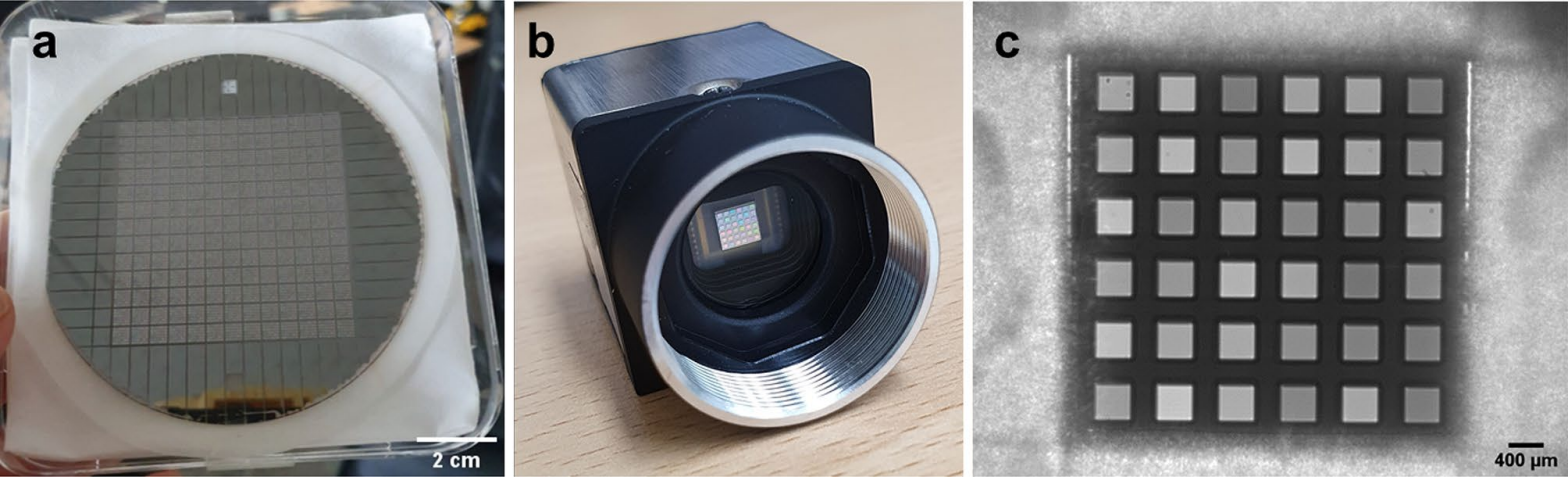
Fabricated MTF filter array. (**a**) 169 identical MTF filter arrays fabricated in a single wafer. (**b**) Photograph of the CMOS image camera with the fabricated MTF filter array. (**c**) Monochrome image of the fabricated MTF filter array illuminated by a halogen light source.

We fabricated 169 identical filter arrays on a single wafer, as shown in Fig. 2a. The filter array is the shape of a 6 × 6 square grid. The size of the square is 400 × 400 μm^2^, and the space between the squares is 300 μm. Accordingly, the size of filter array is 4.5 × 4.5 mm^2^. To fabricate filter arrays, we use TiO_2_ and SiO_2_ as a high and low refractive index materials, respectively. The refractive indices for TiO_2_ and SiO_2_ are approximately 2.6 and 1.45 at 600 nm, respectively.

Unlike etalon filters that were fabricated by changing the thickness of interspacing dielectric layers^20,29^, we fabricated the MTF filters by changing the number of layers and thicknesses of layers. An MTF filter with a unique transmission function can be obtained by repeatedly alternating the two materials and depositing them with different thicknesses. 36 MTF filters with unique transmission functions were fabricated by selectively omitting certain layers of different MTF filters using shadow masks during the deposition of the MTF filter array. As shown in Fig. 1b, the upper and lower layers sum up to form one layer with a different thickness by omitting an intermediate layer. The designed thicknesses of layers for MTF filters are shown in Supplementary Information Table S1. The deposition process for creating filter arrays follows.

TiO_2_ and SiO_2_ films were deposited onto a borosilicate glass wafer whose refractive index is approximately 1.472 at 588 nm. In order to distinguish where the material should be deposited, shadow masks were used. The desired thickness of TiO_2_ is deposited on the desired locations using direct current (DC) magnetron sputter. For TiO_2_ deposition, a Ti target was sputtered in a mixture of argon (Ar) and oxygen (O_2_). The mixture gas flow of 188 sccm of Ar and 12 sccm of O_2_ was utilized and the DC power was 700 W. The TiO_2_ deposition is performed only on the desired region designated by the shadow mask. Then, the shadow mask is changed, with different patterns on the other mask, and we deposit SiO_2_ at the intended thickness. Radio frequency (RF) magnetron sputter was used for the SiO_2_ deposition. A Si target was sputtered in a mixture of Ar and O_2_. The mixture gas flow of 185 sccm of Ar and 15 sccm of O_2_ was utilized. The RF power was 300 W. The deposition is repeated 17 additional times by changing the shadow mask and alternating between TiO_2_ and SiO_2_. Hence, we conducted ten individual depositions of TiO_2_ and nine individual depositions of SiO_2_. The number of shadow masks used in depositions was 19. After completing thin film deposition, we coated the surface of thin films with a photoresist. Germanium (Ge) was deposited over the entire wafer area using an e-beam evaporator. Lift-off of the photoresist was performed by soaking the deposited wafer in acetone. When the photoresist was washed away, Ge deposited on the top of the photoresist was lifted off and washed. After lift-off, a square grid of Ge with the size of 400 μm and spacing of 300 μm was formed. The Ge grid was formed to separate MTF filters and prevent incident lights from entering among MTF filters. The wafer cleaning process was then performed, and, finally, the wafer was diced to produce MTF filter arrays.

Unlike the previous work in that SiNx was used as the high refractive index material to fabricate an MTF filter array^13^, we used TiO_2_ as the high refractive index material and could reduce the number of layers for realizing the unique transmission functions. In addition, using stencil lithography, MTF filter arrays could be fabricated in a simplified process that does not involve an etching process.

The MTF filter array-based spectrometer was built by attaching the fabricated MTF filter array to the front of a CMOS monochrome camera, as shown in Fig. 2b. Figure 2c is a monochrome image of the fabricated MTF filter array illuminated by a halogen light source. The image was taken by the CMOS monochrome camera, whose number of pixels is 1280 × 1024. As shown in Fig. 2c, we measure uniform intensity using pixels under a single MTF filter. Also, the pixels have unique intensity according to the MTF filter. Using these unique intensities from MTF filters, we can reconstruct the spectrum of unknown incident light.

### Spectral reconstruction experiments

Here, we address the spectral resolvability of the MTF filter array-based computational spectrometer. An unknown spectrum, ${\mathbf{x}}$, consisting of 350 (*N* = 350) spectral components from wavelengths ranging 500 to 849 nm, is retrieved using measured intensities ${\mathbf{y}}$ with size 36 (*M* = 36). For retrieving the unknown spectrum, we use a sparse representation-based *l*_1_-norm minimization problem. ${\mathbf{x}}$ can be represented as the multiplication of a sparsifying basis ${\mathbf{G}} \in {\mathbb{R}}^{N \times N}$ and a sparse signal ${\mathbf{s}} \in {\mathbb{R}}^{N \times 1} ,$ i.e., ${\mathbf{x = Gs}}.$ Then, Eq. (1) becomes ${\mathbf{y = RGs}}.$ The solution of the sparse signal, ${\hat{\mathbf{s}}}$, can be retrieved by solving the following minimization problem with nonnegativity constraints:

3 $$\mathop {\min }\limits_{{\mathbf{s}}} \left\| {{\mathbf{y - RGs}}} \right\|_{2}^{2} + \gamma \left\| {\mathbf{s}} \right\|_{1} \;{\text{subject to}}\;s_{k} \ge 0\;{\text{for}}\;k = 1,2, \ldots ,N,$$

where $\gamma$ is the non-negative regularization parameter and $\left\| {\mathbf{s}} \right\|_{p}$ is defined as $\left( {\sum\nolimits_{k = 1}^{N} {\left| {s_{k} } \right|^{p} } } \right)^{{{1 \mathord{\left/ {\vphantom {1 p}} \right. \kern-\nulldelimiterspace} p}}} .$ We use the collection of Gaussian distribution functions for the sparsifying basis ${\mathbf{G}}$^4^. The linear combination of Gaussian distribution functions represents the line shape of the spectrum. The retrieved spectrum ${\hat{\mathbf{x}}}$ is ${{\mathbf{G}}{\hat{\mathbf{s}}}}.$ There exist open-source program that can be easily accessed to solve the numerical optimization problem^30,31^. In this work, we use the l1_ls package^31^ to solve the problem. All the spectral reconstructions were done in MATLAB R2017b with an Intel Core i7-5820 K CPU computer. The reconstruction of a single spectrum was done within ~ 0.1 s.

Before conducting spectral reconstructions, we first measured the transmission functions of MTF filters. A beam from the halogen light source (KLS-150H-LS-150D, Kwangwoo) was fed into a monochromator (MMAC-200, Mi Optics). From the monochromator, a monochromatic light with an FWHM of 4 nm was generated. After passing through a collimator, the collimated monochromatic light was fed into the CMOS monochrome camera (EO-1312M, Edmund optics). Using the CMOS camera, we measured the light intensities with and without the MTF filter array. Then, the transmission $T$ of *i*-th filter at wavelength $\lambda_{k}$ is calculated by:

4 $$T_{i} (\lambda_{k} ) = \frac{{IWF_{i} (\lambda_{k} ) - BI_{i} (\lambda_{k} )}}{{IWOF_{i} (\lambda_{k} ) - BI_{i} (\lambda_{k} )}},$$

where $IWF_{i} ,IWOF_{i} ,$ and $BI_{i}$ are intensity with *i*-th filter, intensity without *i*-th filter, and background intensity, respectively. Using the monochromator, we could generate series of monochromatic light at the peak locations from 500 to 849 nm with the step of 1 nm. We captured 350 pairs of monochrome images with and without filters in the wavelength range of 500 to 849 nm. Using Eq. (4), we could obtain transmission functions of 36 MTF filters. The transmission functions were calibrated by element-wise multiplication of the spectral response of the CMOS image sensor as shown in Fig. 1e.

**Figure 3:**
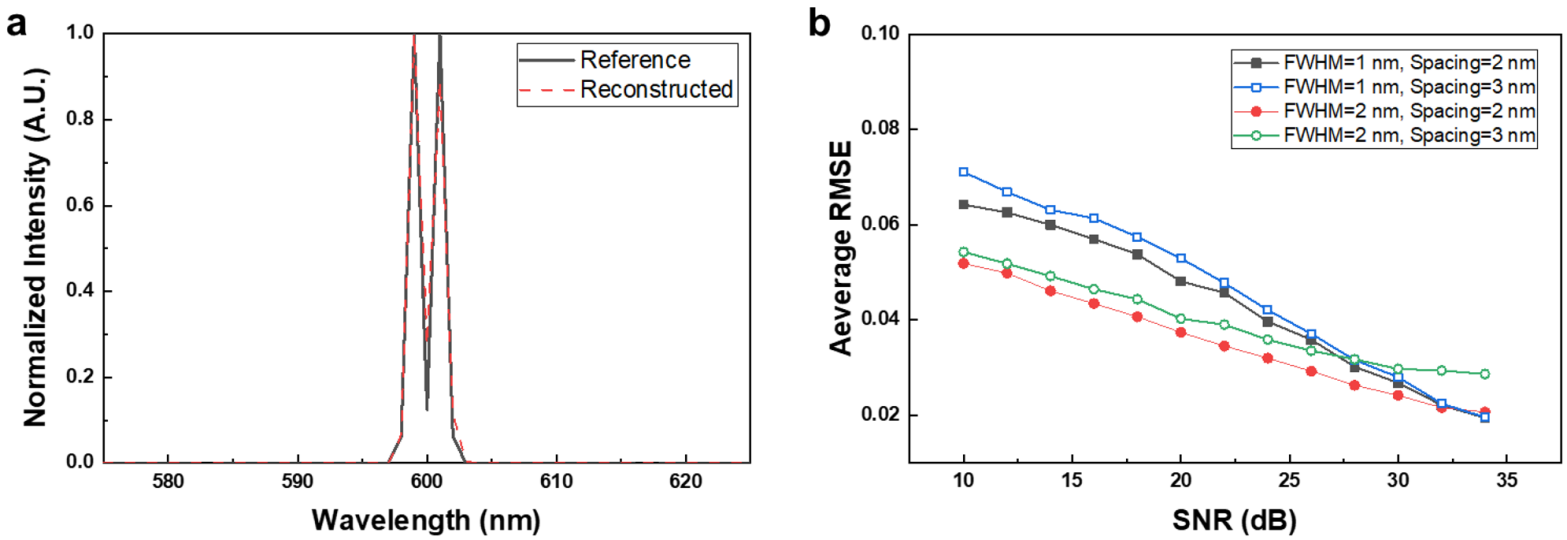
Simulation result of dual-peak spectra reconstructions using MTF filter array. (**a**) An example of dual-peak spectra with an FWHM of 1 nm with 2 nm apart. (**b**) Reconstruction performances of dual-peak spectra with respect to signal-to-noise ratios.

To analyze the dual-peak resolution of the fabricated MTF filter array spectrometer, we conducted simulations of dual-peak spectra reconstructions. Figure 3a shows an example of a dual-peak spectrum. The recovery performances were investigated for noisy environments ranging from 10 to 35 dB of signal-to-noise ratios (SNRs), more details are in the Methods section. The root mean squared error (RMSE), which is defined as $\sqrt {{{\left\| {{\mathbf{x}}_{refer} - {\mathbf{x}}_{recon} } \right\|_{2}^{2} } \mathord{\left/ {\vphantom {{\left\| {{\mathbf{x}}_{refer} - {\mathbf{x}}_{recon} } \right\|_{2}^{2} } N}} \right. \kern-\nulldelimiterspace} N}} ,$ was used to evaluate the performances. The result of dual-peak spectra reconstructions with respect to SNRs is shown in Fig. 3b. We considered four kinds of dual-peak spectra. The FWHMs of a peak were 1 and 2 nm, respectively and the gaps between peaks were 2 and 3 nm, respectively. For each kind of dual-peak, spectra were created by changing the location of dual-peak in the wavelength range of 500 to 849 nm. The reconstructions were performed on all these spectra, and RMSEs were calculated. We averaged the RMSEs and regarded the average RMSE value as the performance of the fabricated MTF filter array to reconstruct dual-peak in noisy environments. As shown in Fig. 3b, the average RMSEs of dual-peak with the FWHM of 1 nm and the gap of 2 nm were 0.0268 for 30 dB, 0.0481 for 20 dB, and 0.0643 for 10 dB, respectively. Similar performances were obtained from the other three kinds of dual-peak spectra. We could find that the fabricated MTF filter array performs well to reconstruct dual-peak spectra in noisy environments.

After analyzing the dual-peak resolution of the MTF filter array in simulations, we tested monochromatic lights by varying the peak wavelength. The CMOS monochrome camera was used for measuring the intensities of test lights. The pixel size of the CMOS image sensor is 5.2 × 5.2 μm. Underneath each filter, there are approximately 60 × 60 pixels. However, considering a case where the layer’s location mismatch may occur during the fabrication process of the MTF filter, we excluded the boundary pixels. The averaged intensities from 30 × 30 pixels at the center of each filter were used for the spectral reconstruction experiments. Using a grating-based spectrometer (Black-Comet, StellarNet), monochromatic lights were measured for use as a reference.

**Figure 4:**
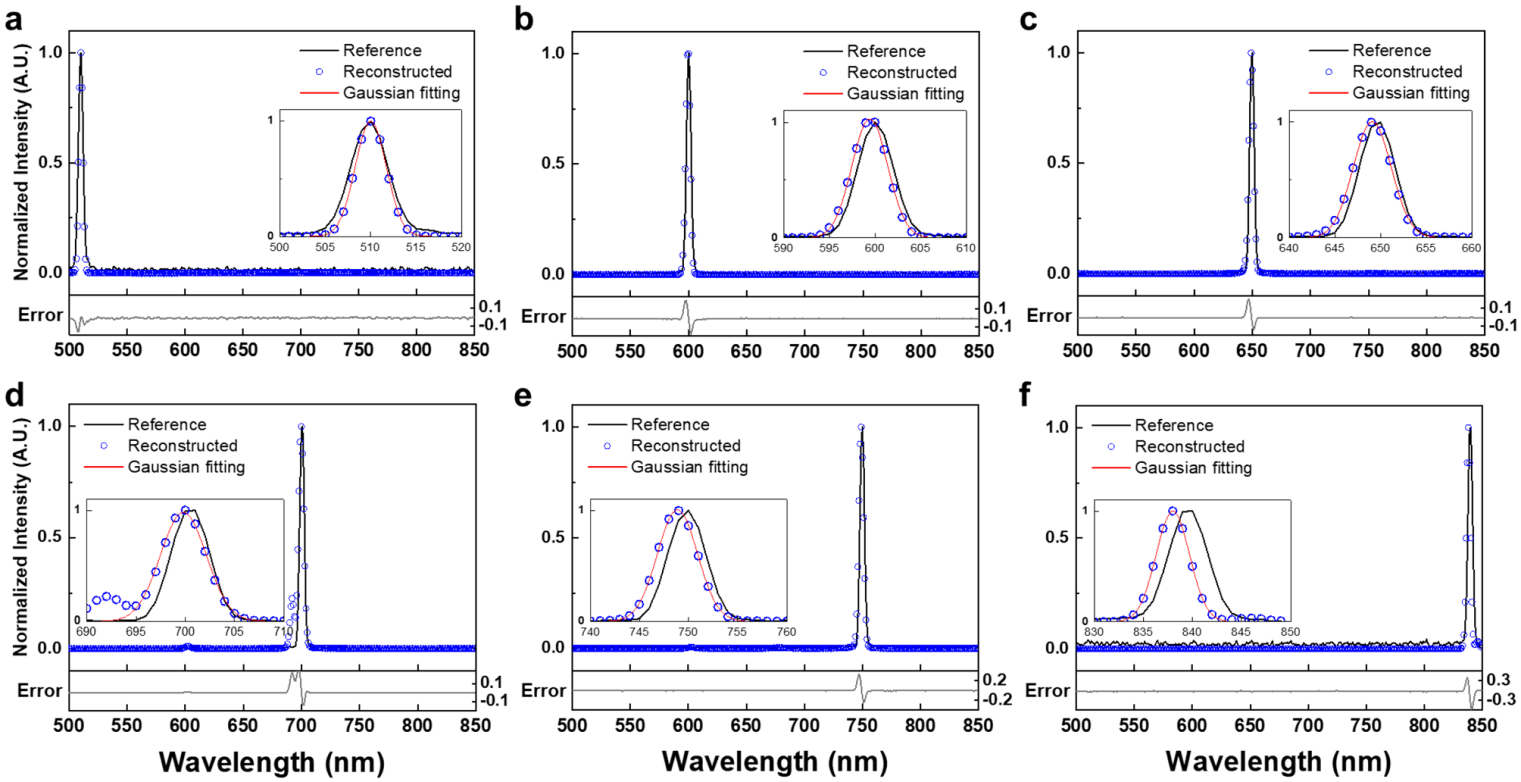
Spectral reconstruction of monochromatic light sources. Monochromatic light with an FWHM of 4 nm at peak wavelengths of (**a**) 510 nm, (**b**) 600 nm, (**c**) 650 nm, (**d**) 700 nm, (**e**) 750 nm, and (**f**) 840 nm. Solid black lines represent reference spectra which are measured by the grating-based spectrometer. Blue circles represent reconstructed spectra using the MTF filter array spectrometer. Solid red lines represent the results of Gaussian fitting. Solid light gray lines represent error between reconstructed and reference spectra.

Figure 4 shows the reconstruction results for the monochromatic light. For ease of comparison, reference spectra and reconstructed spectra are normalized. Solid black lines and blue circles in Fig. 4 represent reference spectra and reconstructed spectra, respectively. Reference spectra have peak wavelengths at 510, 600, 650, 700, 750 and 840 nm with FWHMs of 4 nm. As depicted in the inset enlarged graph, the reconstructed spectra using the MTF filter array spectrometer matched the reference spectra. More specifically, differences of peak wavelengths between reference and reconstructed spectra were within 2 nm. The RMSEs were 0.023, 0.023, 0.021, 0.035, 0.035, and 0.061, for wavelengths 510, 600, 650, 700, 750, and 840 nm, respectively, as shown in Fig. 4a–f. Table 1 presents the evaluation of monochromatic light reconstructions using Gaussian fittings. Over monochromatic lights, peak shifts and FWHMs were within 2 nm and 5.5 nm, respectively. Spectral reconstruction performance seems to degrade in the long-wavelength range due to the low spectral response of the CMOS image sensor and the monotonous spectral features of MTF filters.

**Table 1:** Evaluation of monochromatic lights reconstructions using the Gaussian curve fitting.

Monochromatic light	510 nm	600 nm	650 nm	700 nm	750 nm	840 nm
Peak Center (nm)	509.995	599.438	649.092	699.808	748.878	838.001
Peak shift (nm)	0.005	0.562	0.908	0.192	1.122	1.999
FWHM (nm)	4.008	4.597	4.887	5.486	4.79	4.003

**Figure 5:**
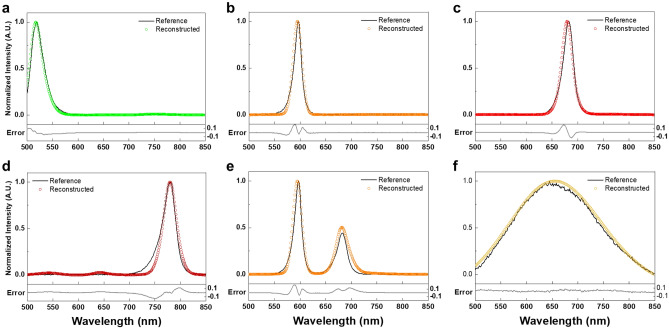
Spectral reconstructions of LEDs and a halogen light source. (**a**) a green LED, (**b**) an orange LED, (**c**) a red LED, (**d**) an infrared LED, (**e**) Combined two LEDs (orange and red), and (**f**) a halogen light source. Solid black lines represent reference spectra which are measured by the grating-based spectrometer. Colored circles represent reconstructed spectra using the MTF filter array spectrometer. Solid light gray lines represent error between reconstructed and reference spectra.

We further explored the performance of the MTF filter array spectrometer using broadband light sources, such as LEDs and a halogen light source. Figure 5 shows the spectral reconstruction results. Solid black lines represent reference spectra, which are measured using the grating-based spectrometer. Colored circles represent reconstructed spectra using the MTF filter array spectrometer. Three single-color visible LEDs and one single-color infrared LED were used for spectral reconstruction experiments, as shown in Fig. 5a–d. A green LED (LED 525E, Thorlabs) with an FWHM of 32 nm was reconstructed with an RMSE of 0.021. An orange LED (LED 600L, Thorlabs) with an FWHM of 12 nm was reconstructed with an RMSE of 0.034. A red LED (LED 680L, Thorlabs) with an FWHM of 16 nm was reconstructed with an RMSE of 0.035. An infrared LED (LED 780E, Thorlabs) with an FWHM of 25 nm was reconstructed with an RMSE of 0.044. Similar to experimental results of monochromatic light, the reconstruction performance is relatively poor for spectrum in the long-wavelength range. In addition, we conducted the spectral reconstruction for combined LEDs (an orange LED and a red LED), as shown in Fig. 5e. A beam splitter is used to measure the light of the combined LED. The combined LED was reconstructed with an RMSE of 0.044. Finally, the spectral reconstruction of the halogen light source was conducted. The halogen light source, with an FWHM of 180 nm, was reconstructed with an RMSE of 0.034, as shown in Fig. 5f. As evidenced by low RMSE values, reconstructed spectra agree well with reference spectra measured by the grating-based spectrometer.

### Computational pinhole spectral imaging

**Figure 6:**
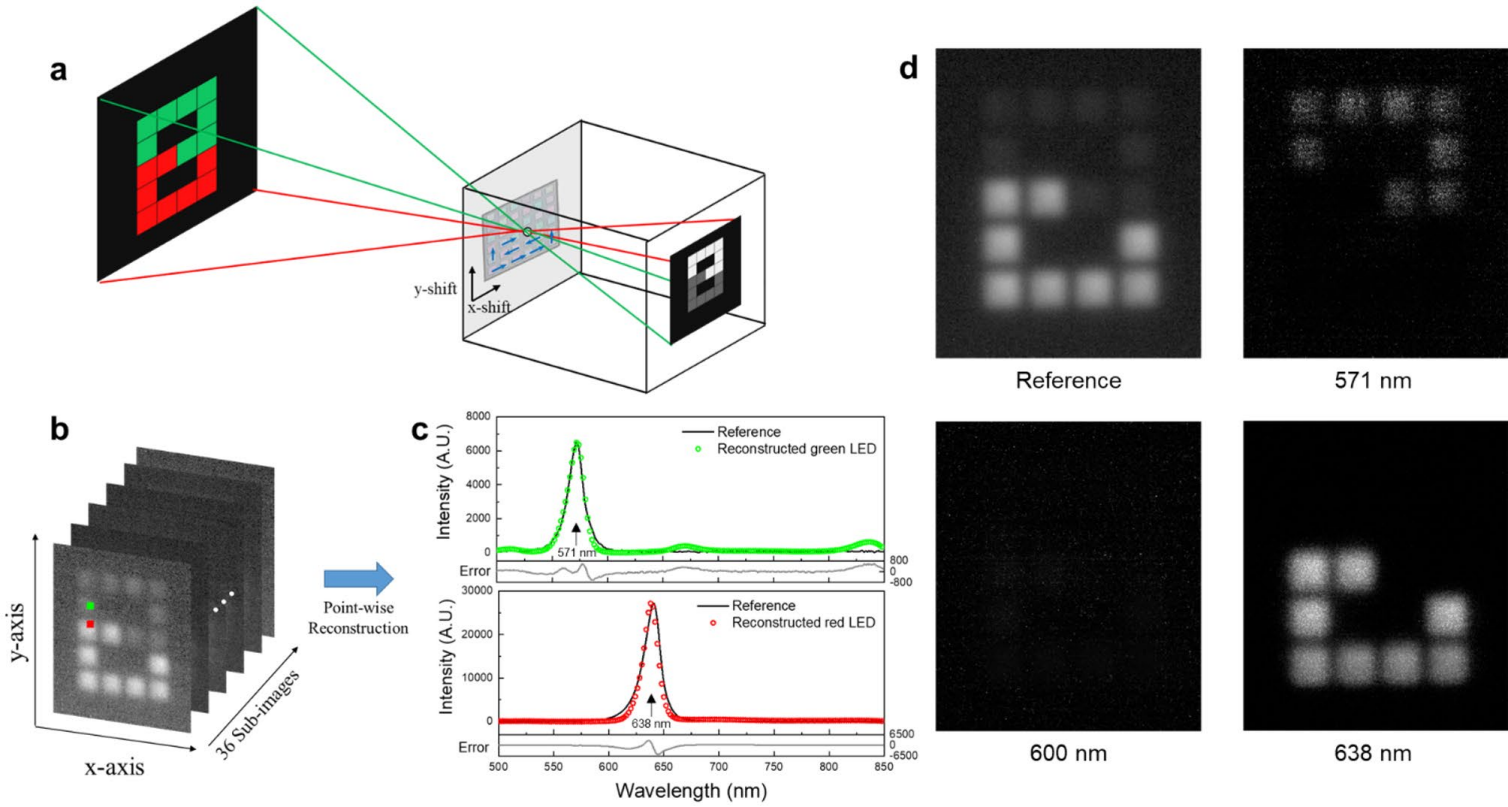
Computational pinhole spectral imaging. (**a**) Schematic of pinhole imaging; The MTF filter array is placed in front of the pinhole camera. A single filter is adjusted to the pinhole and the filtered image is acquired. By changing filters, 36 filtered images are obtained. (**b**) 36 filtered images of 8 × 8 LED matrix showing the number “8”. The upper part consists of green LEDs and the lower part consists of red LEDs. (**c**) Point-wise spectral reconstruction: a pixel of a green LED block and a red LED block which are denoted in (**b**). Solid light gray lines represent error between reconstructed and reference. (**d**) Monochrome image of reference and reconstructed monochrome images at 571, 600 and 638 nm, respectively.

Furthermore, we demonstrated spectral imaging using the MTF filter array. As shown in Fig. 6a, the pinhole imaging system was constructed by combining a pinhole (Edmund optics), whose aperture diameter is 150 μm, with the monochrome CMOS image camera. The MTF filter array was placed in front of the pinhole. A single filter was adjusted to the pinhole to allow an incident image to pass through the filter and pinhole, and the filtered image was measured by the CMOS image sensor. By changing filters using a linear translation stage (Newport), 36 filtered images are obtained. Bi-Color 8 × 8 LED matrix (Adafruit) was used to generate a target. We made a small display by connecting the LED matrix to an Arduino Uno (Arduino) and by controlling the color of the 64 blocks. The number “8” was represented by the LED cube. The upper blocks consist of green LEDs, and the lower blocks consist of red LEDs. Figure 6b shows a stack of the filtered 36 sub-images. A 1280 × 1024 size image was reduced to a sub-image size of 350 × 300 by discarding unnecessary pixels. Thus, a data cube with 350 × 300 × 36 in size was obtained. Spectral reconstruction was performed for each pixel, and the data cube was restored with a size of 350 × 300 × 350. It took ~ 1.8 h to reconstruct the data cube. As shown in Fig. 6c, the reference spectra measured by the grating-based spectrometer are shown in solid black lines. As denoted pixels in Fig. 6b, the reconstructed spectra of a pixel in the green LED block and a pixel in the red LED block are represented in Fig. 6c as green circles and red circles, respectively. The RMSE was calculated after normalizing reference spectra and reconstructed spectra. The green LED with an FWHM of 15 nm was reconstructed with an RMSE of 0.0315. The red LED with an FWHM of 20 nm was reconstructed with an RMSE of 0.0370. Figure 6d shows the monochrome image of reference and reconstructed monochrome images at 571, 600, and 638 nm. The pinhole imaging system also measured the monochrome image of reference without the MTF filter array. Since the spectral component of the red LED does not exist at 571 nm, only the upper blocks of the number “8” are shown in the reconstructed monochrome image at 571 nm. On the other hand, only the lower blocks of the number “8” are shown in the reconstructed monochrome image at 638 nm, where the spectral component of the green LED does not exist. Finally, nothing is displayed in the reconstructed monochrome image at 600 nm, where spectral components of the green and red LED do not exist.

As proof-of-principle of the spectral imaging, we implemented the spectral scanning method on the pinhole imaging system. While reconstructed spectra of the pinhole spectral imaging match well with reference spectra, there are improvements to consider. In the spectral scanning method, the data cube acquisition time is long so that spectral smearing may occur in the case of a moving target. The non-scanning method, such as in snapshot spectral imaging systems, can solve these problems by acquiring the data cube in a single exposure. We assume that it is possible to construct a snapshot spectral imaging system combining the MTF filter array and a thin observation module by bound optics (TOMBO)^32–34^ structure. This spectral imaging system requires a microlens array and a single separator but does not need the MTF filter to be as small as pixel size. Rather, the MTF filter should be made large so that many pixels are underlying the filter. The MTF filter array can be fabricated in scalable using stencil lithography techniques according to the spatial resolution of the spectral imaging system.

## Discussion

In conclusion, we mass-produced MTF filter arrays using stencil lithography and experimentally demonstrated the spectral resolvability of an MTF filter array-based computational spectrometer. 169 identical filter arrays with 36 MTF filters were fabricated on a single wafer. Although the MTF filter size was larger than that of the photonic crystal slabs^14,16^, it can be improved to a smaller size by using advanced lithography techniques and facilities. In addition, by using a higher refractive index material, the number of layers of the MTF filter can be reduced so that manufacturing efficiency can be improved.

Using the random spectral features of MTF filters and numerical optimization techniques, we recover varied spectra from the visible range to the near-infrared range (500 to 849 nm) with 1 nm spacing. The spectral reconstruction performance in the near-infrared range is relatively inferior to the visible range, but it can be further improved by using a CMOS image sensor with a high spectral response in the near-infrared region. Also, computational spectral imaging with the MTF filter array was demonstrated using the spectral scanning method. The reconstructed data cube was found to match well with spatial and spectral references. However, to use the spectral imaging system in mobile applications, a shorter data cube acquisition time is required. By utilizing the TOMBO structure with the MTF filter array, it is possible to construct a snapshot spectral imaging system that has a short acquisition time.

Finally, the production of the MTF filter arrays is an important step towards the industrialization and practical uses of computational spectrometers. This study will be helpful for computational spectroscopy to be used in various applications where compact size, high resolution, and wide working range are required.

## Methods

### Simulation details

Using Gaussian distribution functions, we generated a dual-peak spectrum **x** as shown in Fig. 3a. The spectrum **x** is computationally measured as **y** by multiplying the sensing matrix **R**, i.e., ${\mathbf{y = Rx}}$. In addition, we made noisy measurement ${\tilde{\mathbf{y}}}$ by adding additive noise **n** as ${\tilde{\mathbf{y}}} = {\mathbf{y}}+ {\mathbf{n}} = {\mathbf{Rx }} + {\mathbf{n}}$. SNR in decibels is defined as $10\log \left( {{{\left\| {\mathbf{x}} \right\|_{2}^{2} } \mathord{\left/ {\vphantom {{\left\| {\mathbf{x}} \right\|_{2}^{2} } {N\sigma^{2} }}} \right. \kern-\nulldelimiterspace} {N\sigma^{2} }}} \right)$ where $\sigma$ is the standard deviation of the noise.

## Supplementary Information

Supplementary Table S1.

## Data Availability

Raw data is available from the corresponding author upon reasonable request.
