# Supplementary material for: Mass production-enabled computational spectrometers based on multilayer thin films
Source: Sci Rep. 2022 Mar 8;12:4053. doi: 10.1038/s41598-022-08037-y (PMC8904474; doi:10.1038/s41598-022-08037-y)
Supplement: Supplementary file 1 — Supplementary Table S1. [file 41598_2022_8037_MOESM1_ESM.docx]

***Supplementary information***

***for***

**Mass production-enabled computational spectrometer based on multilayer thin films**

**Cheolsun Kim, Pavel Ni,** **Kang Ryeol Lee and Heung-No Lee***

**Table S1.** The designed thicknesses of layers for MTF filters

| **Unit (nm)** | 1^st^ layer | 2 | 3 | 4 | 5 | 6 | 7 | 8 | 9 | 10 | 11 | 12 | 13 | 14 | 15 | 16 | 17 | 18 | 19 |
| --- | --- | --- | --- | --- | --- | --- | --- | --- | --- | --- | --- | --- | --- | --- | --- | --- | --- | --- | --- |
| **Top**  **(air)** | TiO_2_ | SiO_2_ | TiO_2_ | SiO_2_ | TiO_2_ | SiO_2_ | TiO_2_ | SiO_2_ | TiO_2_ | SiO_2_ | TiO_2_ | SiO_2_ | TiO_2_ | SiO_2_ | TiO_2_ | SiO_2_ | TiO_2_ | SiO_2_ | TiO_2_ |
| Filter  1 | 86 | 83 | 86 | 188 | 92 | 109 | 204 | 150 | 232 | 61 | 137 | 165 | 163 | 215 | 75 | 110 | 50 | 241 | 204 |
| Filter  2 | 86 | 83 | 86 | 188 | 92 | 109 | 204 | 150 | 232 | 61 | 137 | 165 | 163 | 215 | 0 | 110 | 0 | 241 | 204 |
| Filter  3 | 86 | 83 | 86 | 188 | 92 | 109 | 204 | 150 | 232 | 61 | 0 | 165 | 163 | 215 | 75 | 0 | 50 | 241 | 204 |
| Filter  4 | 86 | 83 | 86 | 0 | 92 | 0 | 204 | 150 | 232 | 61 | 137 | 165 | 163 | 215 | 75 | 110 | 50 | 241 | 204 |
| Filter  5 | 86 | 83 | 86 | 188 | 92 | 109 | 0 | 150 | 232 | 61 | 137 | 165 | 0 | 215 | 75 | 110 | 50 | 241 | 204 |
| Filter  6 | 86 | 83 | 86 | 188 | 92 | 109 | 204 | 150 | 232 | 61 | 137 | 165 | 163 | 215 | 75 | 0 | 0 | 241 | 204 |
| Filter  7 | 0 | 83 | 86 | 188 | 92 | 109 | 204 | 150 | 232 | 0 | 137 | 165 | 163 | 215 | 75 | 110 | 50 | 241 | 204 |
| Filter  8 | 86 | 83 | 86 | 188 | 92 | 109 | 204 | 150 | 232 | 61 | 137 | 165 | 0 | 215 | 75 | 0 | 50 | 241 | 204 |
| Filter  9 | 86 | 83 | 86 | 188 | 92 | 109 | 204 | 0 | 232 | 61 | 137 | 165 | 163 | 215 | 75 | 110 | 50 | 0 | 204 |
| Filter 10 | 86 | 83 | 86 | 188 | 92 | 109 | 0 | 150 | 232 | 61 | 137 | 165 | 163 | 0 | 75 | 110 | 0 | 241 | 204 |
| Filter 11 | 86 | 83 | 86 | 188 | 0 | 109 | 204 | 150 | 232 | 61 | 0 | 165 | 163 | 215 | 75 | 110 | 50 | 241 | 0 |
| Filter 12 | 86 | 83 | 86 | 0 | 92 | 0 | 204 | 150 | 232 | 61 | 137 | 165 | 163 | 215 | 75 | 110 | 50 | 241 | 0 |
| Filter 13 | 86 | 83 | 86 | 188 | 92 | 109 | 204 | 150 | 0 | 61 | 0 | 165 | 163 | 0 | 75 | 110 | 50 | 241 | 204 |
| Filter 14 | 86 | 83 | 86 | 188 | 92 | 109 | 0 | 150 | 0 | 61 | 0 | 165 | 163 | 215 | 75 | 110 | 50 | 241 | 204 |
| Filter 15 | 0 | 83 | 86 | 188 | 92 | 109 | 204 | 150 | 232 | 61 | 137 | 165 | 0 | 215 | 75 | 110 | 50 | 241 | 0 |
| Filter 16 | 86 | 83 | 86 | 188 | 92 | 109 | 204 | 150 | 232 | 61 | 0 | 165 | 163 | 0 | 75 | 110 | 50 | 0 | 204 |
| Filter 17 | 86 | 0 | 86 | 188 | 92 | 109 | 204 | 150 | 232 | 61 | 137 | 165 | 163 | 215 | 0 | 110 | 0 | 241 | 204 |
| Filter 18 | 86 | 83 | 86 | 0 | 92 | 109 | 204 | 150 | 232 | 61 | 137 | 0 | 0 | 215 | 75 | 110 | 50 | 241 | 204 |
| Filter 19 | 86 | 83 | 86 | 0 | 92 | 0 | 204 | 150 | 232 | 0 | 0 | 165 | 163 | 215 | 75 | 110 | 50 | 241 | 204 |
| Filter 20 | 86 | 83 | 86 | 0 | 92 | 0 | 204 | 150 | 0 | 0 | 137 | 165 | 163 | 215 | 75 | 110 | 50 | 241 | 204 |
| Filter 21 | 86 | 83 | 0 | 188 | 92 | 109 | 204 | 150 | 232 | 61 | 137 | 165 | 163 | 215 | 75 | 0 | 0 | 0 | 204 |
| Filter 22 | 86 | 83 | 86 | 0 | 92 | 109 | 204 | 0 | 232 | 61 | 137 | 165 | 163 | 215 | 0 | 0 | 50 | 241 | 204 |
| Filter 23 | 86 | 83 | 86 | 188 | 92 | 109 | 0 | 150 | 232 | 61 | 0 | 0 | 0 | 215 | 75 | 110 | 50 | 241 | 204 |
| Filter 24 | 86 | 83 | 86 | 0 | 92 | 109 | 204 | 0 | 232 | 61 | 137 | 0 | 163 | 0 | 75 | 110 | 50 | 241 | 204 |
| Filter 25 | 86 | 0 | 86 | 188 | 0 | 109 | 0 | 150 | 232 | 61 | 137 | 165 | 0 | 215 | 75 | 110 | 50 | 241 | 204 |
| Filter 26 | 86 | 83 | 86 | 188 | 0 | 109 | 204 | 0 | 232 | 0 | 137 | 165 | 163 | 215 | 75 | 110 | 50 | 0 | 204 |
| Filter 27 | 86 | 83 | 86 | 188 | 0 | 109 | 0 | 150 | 232 | 61 | 137 | 165 | 163 | 215 | 75 | 110 | 0 | 241 | 0 |
| Filter 28 | 86 | 83 | 86 | 188 | 92 | 109 | 204 | 0 | 232 | 0 | 137 | 0 | 163 | 0 | 75 | 0 | 50 | 241 | 204 |
| Filter 29 | 0 | 0 | 86 | 0 | 92 | 109 | 204 | 150 | 232 | 61 | 137 | 165 | 163 | 215 | 75 | 110 | 0 | 0 | 204 |
| Filter 30 | 86 | 83 | 86 | 0 | 92 | 0 | 0 | 150 | 0 | 61 | 137 | 165 | 0 | 215 | 75 | 110 | 50 | 241 | 204 |
| Filter 31 | 86 | 83 | 86 | 188 | 0 | 0 | 204 | 150 | 232 | 0 | 0 | 165 | 163 | 215 | 75 | 110 | 50 | 241 | 0 |
| Filter 32 | 86 | 83 | 0 | 188 | 92 | 109 | 204 | 150 | 232 | 61 | 0 | 0 | 0 | 215 | 75 | 110 | 50 | 0 | 204 |
| Filter 33 | 86 | 83 | 86 | 188 | 0 | 109 | 204 | 0 | 0 | 61 | 137 | 165 | 163 | 215 | 0 | 110 | 50 | 241 | 0 |
| Filter 34 | 0 | 83 | 0 | 188 | 0 | 109 | 204 | 0 | 232 | 61 | 137 | 165 | 163 | 215 | 75 | 110 | 50 | 241 | 0 |
| Filter 35 | 86 | 83 | 0 | 188 | 0 | 109 | 0 | 0 | 232 | 61 | 137 | 165 | 163 | 0 | 75 | 110 | 50 | 241 | 204 |
| Filter 36 | 86 | 83 | 86 | 188 | 0 | 109 | 204 | 150 | 0 | 61 | 137 | 165 | 163 | 0 | 75 | 0 | 0 | 241 | 204 |
